# A novel molecular link between HOXA9 and WNT6 in glioblastoma identifies a subgroup of patients with particular poor prognosis

**DOI:** 10.1002/1878-0261.12633

**Published:** 2020-05-06

**Authors:** Céline S. Gonçalves, Ana Xavier‐Magalhães, Eduarda P. Martins, Afonso A. Pinto, Manuel Melo Pires, Célia Pinheiro, Rui M. Reis, Nuno Sousa, Bruno M. Costa

**Affiliations:** ^1^ Life and Health Sciences Research Institute (ICVS) School of Medicine University of Minho Braga Portugal; ^2^ ICVS/3B’s ‐ PT Government Associate Laboratory Braga/Guimarães Portugal; ^3^ Department of Neurosurgery Hospital Escala Braga Braga Portugal; ^4^ Neuropathology Unit Department of Neurosciences Porto Portugal; ^5^ Neurosurgery Centro Hospitalar do Porto Porto Portugal; ^6^ Molecular Oncology Research Center Barretos Cancer Hospital Barretos Brazil

**Keywords:** DNA methylation, glioma, homeobox, HOX, regulation, WNT pathway

## Abstract

Despite much effort to improve treatments, patients with malignant glioma still present a very poor prognosis that has not changed significantly in the last decades. In this context, it is crucial to better understand glioma pathogenesis to identify new molecular prognostic subgroups and therapeutic targets. WNT6 was recently identified as a new oncogenic molecule in glioblastoma (GBM), with prognostic value in patients, but the mechanisms underlying WNT6 aberrant expression in glioma are still unknown. *WNT6* was overexpressed in a subset of gliomas independently of IDH mutations, 1p/19q codeletion status, and *WNT6* gene copy number. Interestingly, *WNT6* expression is associated with the DNA methylation levels of particular CpG regions at both the *WNT6* promoter and the gene body in glioma patient samples. HOXA9, a transcription factor previously associated with poorer clinical outcome in GBM, was identified as a novel transcriptional regulator of *WNT6*, activating the WNT/β‐catenin pathway *in vitro* and *in vivo*. In various cohorts of glioma patients, mRNA levels of *WNT6* and *HOXA9* were significantly correlated, extending our *in vitro* and *in vivo* findings into the clinical setting. Interestingly, this novel molecular link between *WNT6* and *HOXA9* was not limited to glioma, as they were co‐expressed also in patients with other tumor types. Clinically, *WNT6* was a prognostic biomarker of shorter survival in GBM, independently of *HOXA9* expression. Concomitant high expression of both *WNT6* and *HOXA9* identified a subgroup of patients with particularly dismal survival. These findings describe novel *WNT6* regulatory mechanisms in GBM, establishing particular DNA methylation patterns and HOXA9 as critical regulators of WNT6 expression in glioma. This HOXA9‐WNT6 molecular link supports WNT signaling in GBM cells and is a powerful prognostic biomarker, highlighting the clinical relevance of this axis in patients. Novel therapies targeting WNT6‐HOXA9 signaling may thus be useful for this deadly disease.

## Graphical abstract

This work highlights DNA methylation and the homeobox HOXA9 protein as critical regulators of WNT6 expression in malignant glioma. The concomitant expression of HOXA9 and WNT6 results in overactivation of the WNT/β‐catenin signaling pathway *in vitro* and *in vivo*, and identifies a subgroup of glioblastoma patients with particularly dismal prognosis, suggesting this HOXA9‐WNT6 link may be an attractive therapeutic target.

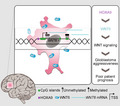

**Abbreviations**
5‐Aza: 5‐aza‐2’‐deoxycytidine
ACC: adrenocortical carcinoma
BLCA: bladder urothelial carcinoma
BRCA: breast invasive carcinoma
CESC: cervical squamous cell carcinoma and endocervical adenocarcinoma
ChIP: chromatin immunoprecipitation
CHOL: cholangiocarcinoma
COAD: colon adenocarcinoma
ES: enrichment score
FDR: false discovery rates
GBM: glioblastoma
GSEA: Gene Set Enrichment Analysis
HB: Hospital Braga
HSA: Hospital Santo António
IF: immunofluorescence
IHC: immunohistochemistry
KPS: Karnofsky performance status
LAML: acute myeloid leukemia
LGG: lower‐grade gliomas
MSigDb: Molecular Signature Database
MSP: methylation‐specific PCR
OS: overall survival
qRT‐PCR: quantitative reverse transcription–PCR
SKCM: skin cutaneous melanoma
TCGA: The Cancer Genome Atlas
TGCT: testicular germ cell tumors
TMZ: temozolomide
WHO: World Health Organization

## 1 Introduction

Gliomas represent 81% of all malignant brain tumors and have been traditionally classified by the World Health Organization (WHO) according to histological features into four malignancy grades (I–IV) (Louis, 2006; Perry and Wesseling, 2016). Glioblastoma (GBM, grade IV) is the most common and lethal glioma in adults, with a median survival of approximately 15 months after diagnosis (Louis *et al.*, 2016; Stupp *et al.*, 2005). Although their clinical response is poor and unpredictable, patients with GBM are still equally treated with a standardized approach that includes surgery, radiotherapy, and chemotherapy, mostly with the alkylating agent temozolomide (TMZ) (Dunn *et al.*, 2012; Weller *et al.*, 2015). In the last years, substantial progress in the understanding of the molecular pathogenesis of gliomas has been achieved (Weller *et al.*, 2015). Although these advances resulted in more refined diagnoses and classifications of glioma tumors, integrating histological and molecular information (e.g., IDH1/2 mutations and 1p/19q codeletion) (Louis *et al.*, 2016), significant improvements in therapies that truly impact on patient outcomes are still lacking.

WNT6, a ligand and activator of the canonical WNT/β‐catenin pathway, was recently described to be overexpressed in GBM, having been associated with patients’ poor prognosis in multiple independent clinical cohorts (Gonçalves *et al.*, 2018). Functionally, WNT6 expression was associated with increased GBM cell viability, proliferation, invasion, migration, resistance to TMZ, and stemness capacity (Gonçalves *et al.*, 2018). *In vivo*, WNT6 accelerated GBM‐associated death in mice. Moreover, WNT6 was shown to activate the WNT, SFK, and STAT pathways, which might be critical effectors of WNT6‐associated aggressiveness in GBM (Gonçalves *et al.*, 2018). Although the relevance of WNT6 overexpression in GBM is now elucidated, the molecular mechanisms underlying WNT6 overexpression in GBM remain essentially unknown. In this context, this study aims to investigate the impact of gene copy number alterations and DNA methylation levels on WNT6 expression, as well as to identify novel transcriptional regulators of WNT6 in GBM. Addressing this critical question may contribute to the development of more rational therapeutic approaches with potential to revert WNT6 activation in the highly aggressive WNT6‐positive GBMs. By integrating *in vitro* and *in vivo* models, and data from patients, this study unravels a novel molecular link between the homeobox *HOXA9* gene and *WNT6* in glioma, which has prognostic relevance in patients with highly aggressive GBMs.

## 2 Materials and methods

### 2.1 TCGA data analysis in glioma patients

The Cancer Genome Atlas (TCGA; https://portal.gdc.cancer.gov/) was used to obtain information about gene expression from lower‐grade glioma (LGG; *n* = 27 for microarray data and *n* = 511 for RNAseq), GBM (*n* = 572 for microarray data and *n* = 161 for RNAseq), adrenocortical carcinoma (*n* = 79), bladder urothelial carcinoma (*n* = 408), breast invasive carcinoma (*n* = 1091), cervical squamous cell carcinoma and endocervical adenocarcinoma (*n* = 304), cholangiocarcinoma (CHOL; *n* = 36), colon adenocarcinoma (*n* = 456), lymphoid neoplasm diffuse large B‐cell lymphoma (*n* = 48), esophageal carcinoma (*n* = 161), head and neck squamous cell carcinoma (*n* = 500), kidney chromophobe (*n* = 65), kidney renal clear cell carcinoma (*n* = 530), kidney renal papillary cell carcinoma (*n* = 288), acute myeloid leukemia (LAML; *n* = 151), liver hepatocellular carcinoma (*n* = 371), lung adenocarcinoma (*n* = 513), lung squamous cell carcinoma (*n* = 501), mesothelioma (*n* = 86), pancreatic adenocarcinoma (*n* = 177), pheochromocytoma and paraganglioma (*n* = 179), prostate adenocarcinoma (*n* = 495), rectum adenocarcinoma (*n* = 166), sarcoma (*n* = 259), skin cutaneous melanoma (SKCM; *n* = 466), stomach adenocarcinoma (*n* = 375), testicular germ cell tumors (TGCT; *n* = 150), thyroid carcinoma (*n* = 502), thymoma (*n* = 119), uterine carcinosarcoma (*n* = 56), and uveal melanoma (*n* = 80). Agilent G4502A 244K data were used for LGG and GBM (*WNT6* and *HOXA9*‐high expression was considered when TCGA level 3 value ≥ 0 [GBM median value] or 3, respectively), while RNAseq data (Illumina HiSeq 2000 Sequencing System) were downloaded for all cancers (*WNT6*‐high expression was considered when TCGA FPKM‐UQ value ≥ 6800 [GBM median value]) (The Cancer Genome Atlas Research Network, 2008). In the Agilent microarray, three probe sets hit *WNT6* gene (A_23_P119916, A_32_P159877, and A_24_P208513) and one hits *HOXA9* (probe A_23_P500998). To prevent duplicated entries from the same patient—when more than one portion per patient was available—the median expression value was used. The provided value was preprocessed and normalized according to ‘level 3’ specifications of TCGA (Gonçalves *et al.*, 2018).

For both LGG and GBM patients, gene copy number, DNA methylation status, and clinical data were also collected (The Cancer Genome Atlas Research Network, 2008).

Gene copy number data from 372 GBM and 514 LGG samples were assessed using Affymetrix Genome‐Wide Human SNP Array 6.0. Gene amplifications or deletions were considered for log_2_ copy number tumor/normal ≥ 0.32 (gene copy number ≥ 2.5) or ≤ −0.42 (gene copy number ≤ 1.5), respectively.

DNA methylation was evaluated using Illumina Infinium Human DNA Methylation 450 array and includes the methylation status of 141 GBM and 516 LGG samples. Twenty‐eight probes spanning from 5000 bp upstream to 5000 bp downstream of the *WNT6* gene were selected, representing a region of ~ 24 kb encompassing three CpG islands (details in Table S1).

Patients’ clinical data (gender, age at diagnosis, Karnofsky performance status (KPS), and days to last follow‐up and death) were obtained from the Biospecimen Core Resources.

### 2.2 Glioma primary samples

Glioma tumor specimens were obtained from patients who performed a craniotomy for tumor removal or stereotaxic biopsy at two different hospitals: Hospital Santo António (HSA, Centro Hospital Porto) and Hospital Braga (HB), Portugal, in a total of 18 GBM and 31 glioma (two WHO grade II, nine grade III, and 20 grade IV) patients, respectively. HSA samples were reserved for DNA‐based studies, while HB samples were used for RNA‐based studies. All samples were transported in dry ice to the laboratory and stored at −80 °C. Only patients with confirmed glial tumor histological diagnosis were included in the study.

### 2.3 Bao and Gill datasets

*WNT6* and *HOXA9* expression RNAseq data from Bao (*n* = 274 gliomas) (Bao *et al.*, 2014) and Gill (*n* = 75 GBM) (Gill *et al.*, 2014) datasets of glioma patients were obtained from the GlioVis data portal (Bowman *et al.*, 2017).

### 2.4 Glioma cell lines

The commercially available pediatric glioma cell lines (UW479, Res186, and KNS42) were cultured in Dulbecco’s modified Eagle’s medium (DMEM)/F12 (Gibco, Grand Island, NY, USA) supplemented with 10% FBS (Biochrom GmbH, Berlin, Germany). Commercially available adult cell lines (SW1783, A172, SNB19, and U87) were purchased from ATCC (Rockville, MD, USA) and cultured in DMEM (Biochrom GmbH) supplemented with 10% FBS. The U87MG cell line was previously (Costa *et al.*, 2010) genetically retrovirally infected with murine stem cell virus (MSCV) containing the *HOXA9* coding region to overexpress this gene (U87‐HOXA9) or with an empty vector (U87‐MSCV, control). U251 cells, which presents endogenous high levels of *HOXA9*, were previously (Pojo *et al.*, 2015) transfected with a shRNA against *HOXA9* to silence its expression (U251 shHOXA9) or with a noneffective shRNA vector (U251 shCtrl). All cells were maintained in a humidified atmosphere at 37 °C and 5% (v/v) CO_2_, and tested monthly for potential mycoplasma contamination.

### 2.5 5‐Aza‐2′‐deoxycytidine (5‐Aza) treatment

Glioma cells were plated in T25 flasks at an initial density of 75 000 cells per flask. Treatment with 5 µm 5‐aza‐2′‐deoxycytidine (5‐Aza) (Sigma‐Aldrich®, St. Louis, MO, USA) or DMSO (Sigma‐Aldrich®) was performed for 72 h with daily renewal. Next, cells were collected by trypsinization, and DNA and RNA were extracted by the TRIzol method (Invitrogen, Grand Island, NY, USA).

### 2.6 Sodium bisulfite treatment

The TRIzol method (Invitrogen) was used to extract DNA from 18 GBM primary tumors and glioma cell lines. After quantification, it was subjected to sodium bisulfite treatment—conversion of unmethylated cytosines to uracil residues, according to manufacturer’s instructions (EZ DNA Methylation‐Gold™ Kit; Zymo Research, Irvine, CA, USA).

### 2.7 Methylation‐Specific PCR (MSP)

*WNT6* DNA methylation was evaluated by MSP on bisulfite‐converted DNAs, using the following sets of primers: unmethylated set, Fwd 5′‐TTTTGTGTTCGGCGTACGT‐3′ and Rev 5′‐AATCTATCCTAAATCCCGAA‐3′; methylated set, Fwd 5′‐TGTTGTTGTTTTTGTGTTTGGTGTAT‐3′ and Rev 5′‐CCCCAATCTATCCTAAATCCCA‐3′. Touchdown MSP was performed (AmpliTaq Gold 360; annealing temperature for unmethylated or methylated primers at 62 °C or 60 °C, respectively—decrement of 1 °C per cycle for 10 cycles—and 52 °C or 50 °C, respectively, for additional 28 cycles). A bisulfite‐treated blood DNA of a control cancer‐free subject (NB599) was used as an unmethylated control for MSPs. A methylated control was obtained by *in vitro* methylation of the same DNA (CpG Methyltransferase M.SssI; New England Biolabs Inc., Ipswich, MA, USA) according to manufacturer’s protocol, followed by sodium bisulfite treatment. All MSP products were loaded onto a 3.5% agarose gel. MSP bands were analyzed using the azurespot 2.0 software (Azure Biosystems, Inc., Dublin, CA, USA) and the automatic lane and band detection, and signal intensity quantification algorithms. A case was considered methylated whenever a clear band corresponding to the methylated reaction was observed and detected by the software (values for the presence of methylated bands ranged between 7718 and 16 533 above the background). A scheme for visualization of the MSP amplification product within the human chromosome 2 (near *WNT6* locus) can be found in Fig. S1.

### 2.8 qRT‐PCR

Total RNA was extracted using the TRIzol method (Invitrogen), and cDNA from 1 µg of the total RNA was synthesized [RT‐Phusion Kit; Thermo Scientific (TM), Waltham, MS, USA] (Goncalves *et al.*, 2015). *WNT6* and *TBP* (reference gene) levels were assessed by quantitative reverse transcription–PCR (qRT‐PCR; KAPA SYBR® FAST qPCR Kit; KAPA BIOSYSTEMS, Wilmington, MA, USA) with the following sets of primers: *WNT6* Fwd 5′–GACGAGAAGTCGAGGCTCTTT–3′ and Rev 5′–CGAAATGGAGGCAGCTTCT–3′; *HOXA9* Fwd 5′–GCCCGTGCAGCTTCCAGTCC–3′ and Rev 5′–GAGCGCGCATGAAGCCAGTTG–3′; and *TBP* Fwd 5′–GAGCTGTGATGTGAAGTTTCC–3′ and Rev 5′–TCTGGGTTTGATCATTCTGTAG–3′. For *WNT6* and *TBP*, the annealing temperature was 60 °C, and 61 °C for *HOXA9*. Levels were determined based on the $2^{-\Delta \Delta C_{t}}$ method, as previously described (Livak and Schmittgen, 2001). The expression data from the Portuguese glioma dataset were log‐transformed (log[relative expression + 1]).

### 2.9 Genomatix analysis

MatInspector from Genomatix software (Cartharius *et al.*, 2005) (http://www.genomatix.de) was used to investigate putative binding sites for transcription factors in the *WNT6* gene. This *in silico* tool identifies transcription factor binding sites in nucleotide sequences based on a large library of weight matrices (Cartharius *et al.*, 2005; Quandt *et al.*, 1995). A perfect match gets a matrix similarity of 1 when the tested sequence corresponds to the most conserved nucleotide at each position of the matrix. A good match to the matrix was considered when matrix similarity > 0.80. The Ci‐value (consensus index) for the matrix represents the degree of conservation of each position within the matrix. A Ci‐value of 100 is reached by a position with total conservation of one nucleotide.

### 2.10 Chromatin Immunoprecipitation (ChIP)

Chromatin immunoprecipitation experiments were done as previously described (Xavier‐Magalhães *et al.*, 2018). The following antibodies were used to immunoprecipitate chromatin: 4 μg anti‐HOXA9 (Santa Cruz Biotechnology, Inc., Dallas, TX, USA), 2 μg anti‐histone H3 (H3; Abcam, Cambridge, UK), and 3 μg anti‐immunoglobulin G (IgG; Sigma‐Aldrich®). DNA amplification was done by qPCR (Maxima SYBR Green; Fermentas, Waltham, MA, USA) with the following set of primers for *WNT6*: Fwd 5′‐CAGGGGCATCAAAGACATTT‐3′ and Rev 5′‐TCAAGAGATCGAGGGGTCAG‐3′, designed to amplify a portion of the promoter region 1000 bp upstream the transcription start region. The annealing temperature was 60 °C. Anti‐IgG and anti‐histone H3 were used as ChIP‐negative and ChIP‐positive controls, respectively. The level of *WNT6* was calculated for each experiment using the$2^{-\Delta \Delta C_{t}}$ method as previously described (Livak and Schmittgen, 2001). Each qPCR experiment was performed thrice, using three biological replicates per cell line.

A scheme for visualization of ChIP PCR amplification product within the human chromosome 2 (near *WNT6* locus) can be found in Fig. S1.

### 2.11 Gene set enrichment analysis (GSEA)

The HOXA9 transcriptome in U87MG GBM cells was previously generated (Pojo *et al.*, 2015). GSEA software (http://www.broad.mit.edu/gsea/) was then used to query transcriptional signatures reminiscent of the U87‐HOXA9 transcriptome (Subramanian *et al.*, 2005). Gene sets from the Molecular Signature Database (MSigDb) C2 collection were selected.

The raw expression data profile (Agilent G4502A 244K) of all GBM patients from TCGA (*n* = 573) was used, employing a continuous phenotype profile to find gene sets from the MSigDb C6 collection that correlates with *WNT6* (gene neighbors). Genes were scored and ranked based on Pearson’s correlation coefficient (Gonçalves *et al.*, 2018). Default options were kept for the remaining parameters. A false discovery rate (FDR) < 0.30 was considered significant.

### 2.12 Immunofluorescence (IF)

For β‐catenin IF (610153; BD Transduction Laboratories, San Jose, CA, USA, 1 : 200), U87‐MSCV and U87‐HOXA9 cells plated on coverslips were fixed in 95% EtOH and 5% acetic acid (v/v), followed by incubation in 1% BSA in PBS‐0.1% Tween for 1 h, and overnight at 4 °C with the primary antibody. Alexa Fluor® 488 Goat Anti‐Mouse IgG [H + L; A‐11001; Thermo Scientific (TM), 1 : 1000] secondary antibody (green) was used. Cell nucleus was stained blue with DAPI (VECTASHIELD® Mounting Medium with DAPI; Vector Laboratories, Inc., Burlingame, CA, USA; 1.5 µg·mL^−1^).

### 2.13 Immunohistochemistry (IHC)

Tissues sections were deparaffinized and rehydrated by xylene and EtOH series, as described in Gonçalves *et al. *(2018). Immunohistochemical staining was performed using the Lab Vision Kit (UltraVision Large Volume Detection System Anti‐polyvalent, HRP) according to the manufacturer’s instructions. WNT6 antibody from Abcam (ab50030; 1 : 450), β‐catenin antibody from BD Transduction Laboratories (610153; 1 : 150), and cyclin D1 antibody from Cell Signaling Technology(R) (Danvers, MA, USA; 2978S; 1 : 100) were used. DAB substrate (DAKO GmbH, Jena, Germany) was used as chromogen, followed by counterstaining with hematoxylin. All antibodies were subjected, by each provider, to quality check assays to ensure peptide specificity (data available in the antibody datasheet or upon request), and have been widely used in the literature.

### 2.14 *In vivo* GBM mouse model

The procedures for the establishment of the subcutaneous mice GBM models were previously described (Pojo *et al.*, 2015). A total of 10 nude mice (athymic nude Foxn1^nu^ male mice, from Harlan Laboratories Inc., Indianapolis, IN, USA) aged 8 months were used. The animals were blindly randomized to be injected with either U87‐MSCV or U87‐HOXA9 GBM cells. All animals included in the study presented similar initial weight and age. Mice were maintained under standard laboratory conditions as previously described (Gonçalves *et al.*, 2018), which included an artificial 12‐h light/dark cycle, controlled ambient temperature (21 ± 1 °C), and a relative humidity of 50–60%. The confirmation of specified pathogen‐free health status of sentinel mice maintained within the same animal room was performed according to FELASA guidelines.

### 2.15 TCF/LEF reporter assay

The Cignal TCF/LEF Reporter Assay Kit (GFP; QIAGEN, Hilden, Germany) was used to quantify the specific activation of β‐catenin‐dependent WNT signaling (canonical pathway) in U87‐MSCV and U87‐HOXA9 cells. The protocol was done following the manufacturer’s recommendations and as previously described (Gonçalves *et al.*, 2018).

### 2.16 Statistical analysis

Correlation values were calculated using Pearson’s or Spearman’s correlation coefficients, according to the normality of the samples (tested by the D’Agostino and Pearson normality test). Levene’s test was used to assess homoscedasticity, and differences between groups were tested by a two‐sided unpaired *t*‐test. Welch’s correction was employed when necessary. graphpad prism 6.01 software was used (GraphPad Software, San Diego, CA, USA).

Survival analyses were performed by log‐rank tests and by multivariable analysis using the Cox proportional hazard model. These analyses were made with spss 22.0 software (SPSS, Inc., Armonk, NY, USA).

For all statistical tests, significance was considered when *P* < 0.05.

### 2.17 Study approval

Written informed consent for investigation purposes was obtained from all patients, according to the Declaration of Helsinki. All procedures were in accordance with institutional ethics standards. No patient information was collected. The ethical approval obtained from Hospital Braga is SECVS 150/2014. All animal procedures were conducted in accordance with the guidelines for the care and use of laboratory animals (European Directive 2010/63/EU) and approved by the Direcção Geral de Alimentação e Veterinária (reference 017761), the competent national authority for animal protection.

## 3 Results

### 3.1 *WNT6* expression in glioma is independent of IDH mutation and 1p/19q codeletion status

**Figure 1:**
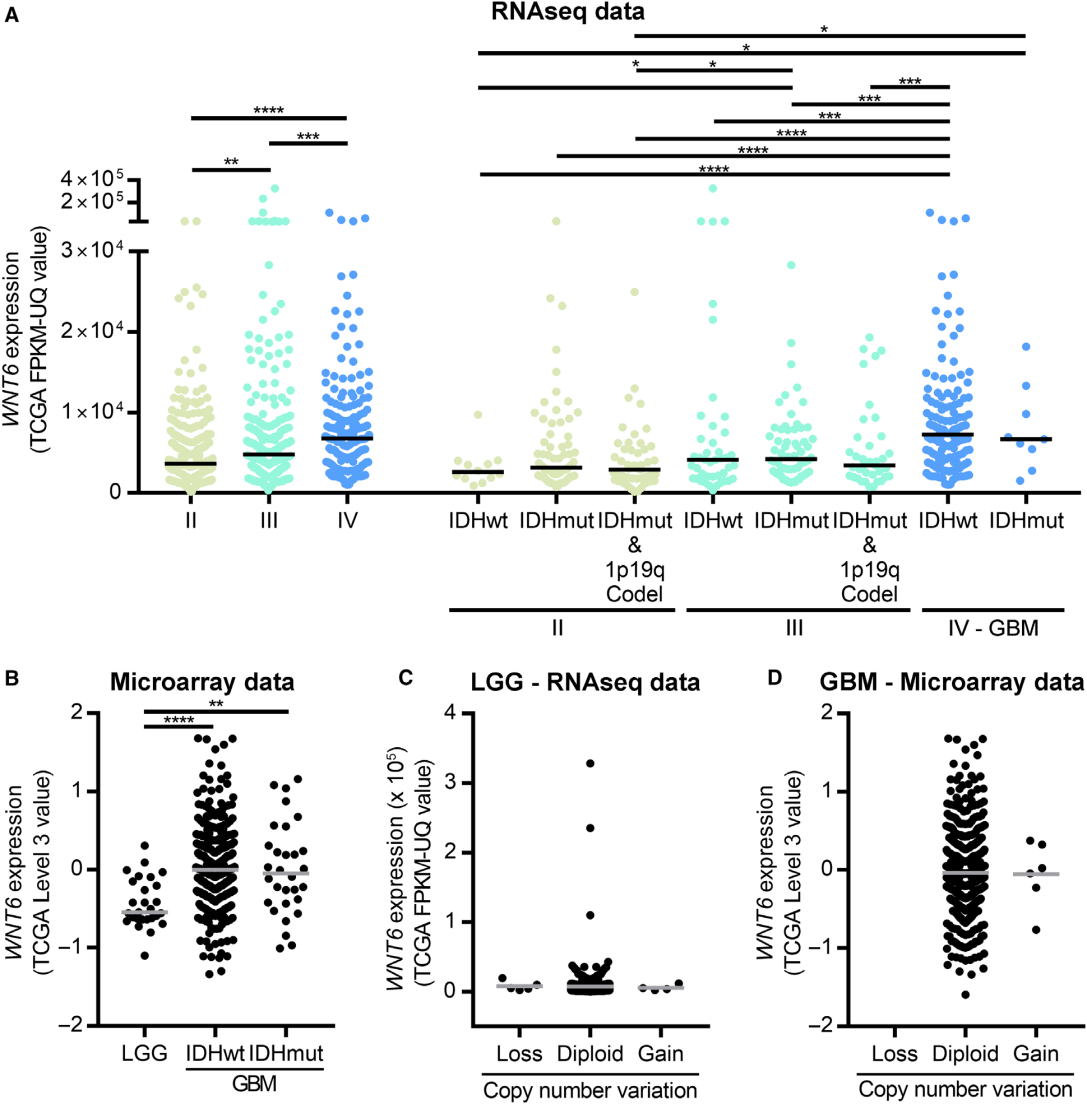
*WNT6* is overexpressed in a subset of gliomas, independently of IDH mutation, 1p/19q codeletion, and *WNT6* gene dosage. (A) RNAseq expression levels of *WNT6* in grade II gliomas (12 IDHwt, 75 IDHmut non‐co‐deleted, and 47 IDHmut codeleted), in grade III gliomas (43 IDHwt, 62 IDHmut non‐co‐deleted, and 37 IDHmut codeleted), and in GBM (143 IDHwt and 9 IDHmut) patients from TCGA. *WNT6* is highly expressed in 23.5%, 31.7%, and 50% of grade II, III, and IV gliomas, respectively; and in 8.3%, 22.7%, and 15% of IDHwt, IDHmut non‐co‐deleted, and IDHmut codeleted grade II; in 25.6%, 27.4%, and 27% of IDHwt, IDHmut non‐co‐deleted, and IDHmut codeleted grade III; and in 52.4% and 44% of IDHwt and IDHmut grade IV (GBM). (B) Microarray expression levels of *WNT6* in 27 lower‐grade gliomas (LGG; grades II and III) and in GBM (368 IDHwt and 30 IDHmut) patients from TCGA. *WNT6* is highly expressed in 7.4% of LGG, in 50% of IDHwt GBM, and in 47% of IDHmut GBM. (C) *WNT6* RNAseq expression levels stratified by gene copy number in LGG patients (five with gene deletion, 500 with no alterations, and four with gene amplification). (D) *WNT6* microarray expression levels stratified by gene copy number in GBM patients (563 with no alterations and six with gene amplification; no deletions were found). *P < 0.05; **P < 0.01; ***P < 0.005; and ****P < 0.0001 (Mann–Whitney test). 1p19q Codel: 1p/19q codeletion.

We previously described that WNT6 expression increases with glioma grade (Gonçalves *et al.*, 2018), but it was still unknown whether this is associated with IDH mutation and/or 1p/19q codeletion status—the two most critical diagnostic molecular factors used in the new 2016 WHO classification of glioma (Louis *et al.*, 2016). Here, using RNAseq data from patients from TCGA (226 grade II, 240 grade III and 161 grade IV gliomas), we found that *WNT6*‐high expression increases with glioma grading (Fig. 1A), independently of IDH mutation or 1p/19q codeletion status [12 IDH‐wildtype (IDHwt), 75 IDH‐mutant (IDHmut) non‐co‐deleted, and 47 IDHmut codeleted grade II; 43 IDHwt, 62 IDHmut non‐co‐deleted, and 37 IDHmut codeleted grade III; and 143 IDHwt and 9 IDHmut GBM]. Concordantly, expanding a similar analysis to the cohort of TCGA glioma patients with microarray data (27 lower‐grade II and III glioma—LGG; and 368 IDHwt and 30 IDHmut GBM) confirmed the overexpression of *WNT6* in higher grades of glioma, both in IDHwt and in IDHmut GBMs (Fig. 1B; of note, due to the limited number of LGG patients with available microarray data [*n* = 27], these cases were not subdivided according to the 2016 CNS classification). Together, these results show that high *WNT6* expression associates with higher glioma grades independently of IDH mutation and 1p/19q codeletion status.

### 3.2 *WNT6* expression is regulated by DNA methylation in gliomas

To understand the mechanisms responsible for *WNT6* overexpression in glioma, we started by investigating copy number alterations of the *WNT6* locus in LGG (*n* = 509) and GBM (*n* = 565) patients from TCGA (Fig. 1C,D). In LGG, only four patients (0.79%) presented *WNT6* gene amplification, while the region encompassing *WNT6* was deleted in five cases (0.98%; Fig. 1C). In GBM, only six patients (1.06%) presented *WNT6* gene amplification (Fig. 1D), and no deletions were observed. Both in LGG and in GBM, the very rare *WNT6*‐amplified cases did not present high expression levels of *WNT6*. These data show that copy number aberration is not a major mechanism mediating *WNT6* overexpression in GBM.

**Figure 2:**
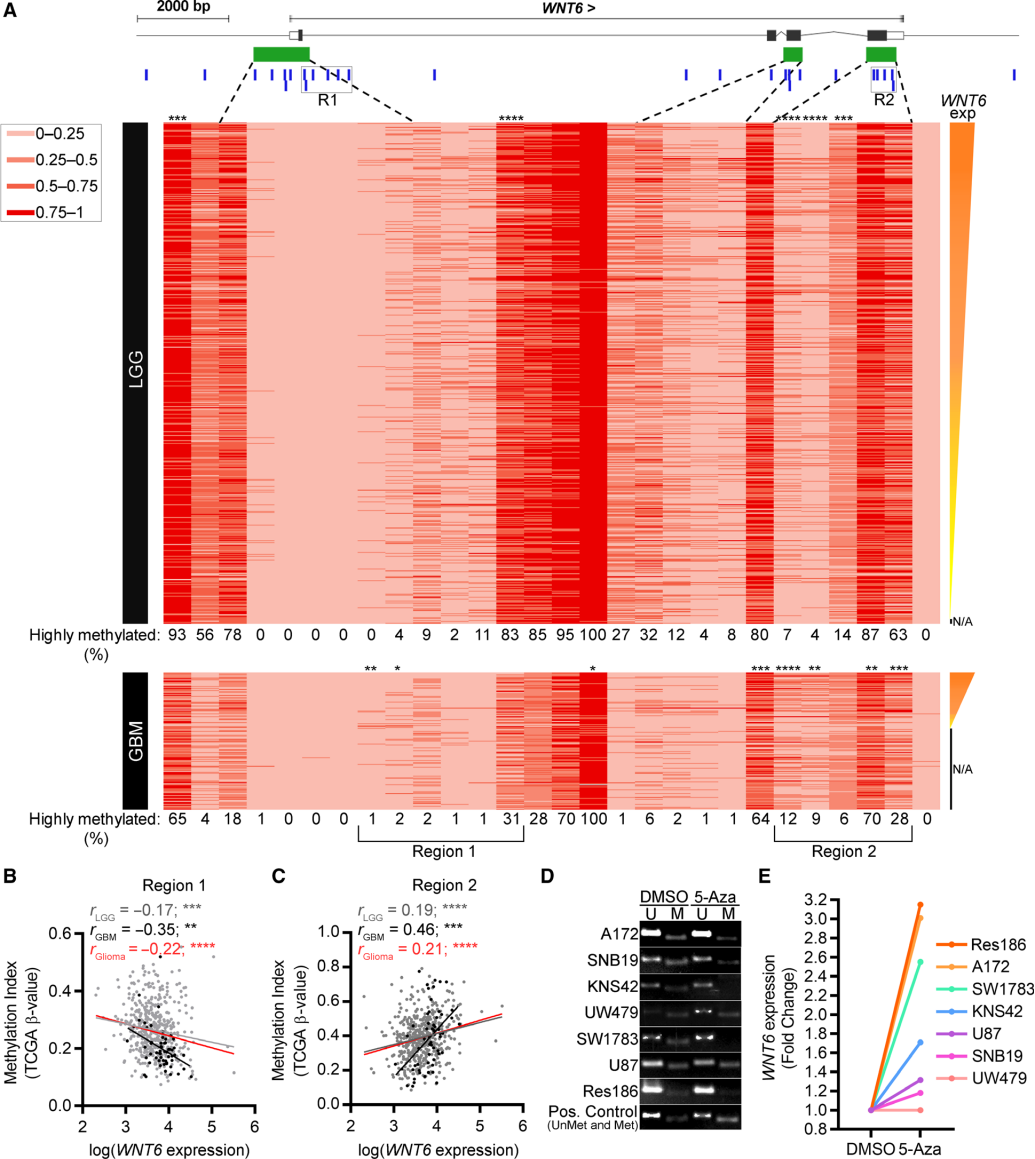
*WNT6* is transcriptionally regulated by DNA methylation in gliomas. (A) Heatmap representation of DNA methylation levels (TCGA β‐values) corresponding to *WNT6* locus in 516 LGG (top) and 141 GBM (bottom) patients from TCGA. Each column corresponds to a probe and each row to a patient. A total of 28 methylation probes (vertical blue bars) were assessed. CpG islands > 300 bp are represented in green. Coding exons are represented by blocks, connected by lines representing introns. White rectangles at the left and right ends represent the 5′ and 3′ UTR, respectively. The methylation color code with TCGA β‐values is shown on the top left. Patients are ranked based on *WNT6* expression (obtained by RNAseq for 511 out of 516 LGGs and 58 out of 141 GBMs), as shown on the right. The percentage of highly methylated cases (TCGA β‐values ≥ 0.5) for each CpG probe are depicted for LGG and GBM below their respective heatmap. Probes whose methylation levels were statistically correlated with *WNT6* expression levels (|*r*|> 0.15 and *P* < 0.05) are marked with * above the respective column (Pearson’s or Spearman’s correlation, according to the normality of the samples, as tested by D’Agostino and Pearson normality test). (B, C) Correlation graphs between *WNT6* expression (log‐transformed TCGA FPKM‐UQ value, RNAseq data) and average DNA methylation index (TCGA β‐values) of the probes from both selected regions (Regions 1 and 2) of 511 LGG (gray dots and linear regression line), 58 GBM (black dots and linear regression line), and 569 gliomas (red linear regression line). (D) MSP functional assessment of WNT6 locus (querying CpGs in Region 1) and (E) *WNT6* expression levels in glioma cell lines treated with DMSO or 5‐Aza. *P < 0.05; **P < 0.01; ***P < 0.005; and ****P < 0.0001; N/A = not available; R1 = Region 1; R2 = Region 2.

We next evaluated *WNT6*’s DNA methylation levels in glioma patients and investigated whether these might be associated with *WNT6* expression levels (Figs 2, S2 and S3). Statistical correlation analyses were performed for each probe of the *WNT6* locus, in 511 LGG and 58 GBM for which both expression (RNAseq data) and DNA methylation data were available (Fig. 2A). Interestingly, the DNA methylation levels of five probes were significantly correlated with *WNT6* expression in LGG patients (two inversely correlated and three positively correlated; Fig. S2A). Similarly, in GBM, significant correlations were observed in eight CpG sites (two inversely correlated and six positively correlated; Fig. S2B). The analysis of the DNA methylation levels of probes neighboring those with significant correlations revealed a particular region located closely downstream of the *WNT6* promoter (Region 1; Fig. 2B) that was significantly inversely correlated with *WNT6* expression, particularly in GBM patients (*r* = −0.35; *P* < 0.01). Interestingly, the DNA methylation levels of a second region encompassing a CpG island within the *WNT6* gene body (Region 2; Fig. 2C) were positively correlated with *WNT6* expression, particularly in GBM patients (*r* = 0.46; *P* < 0.001). Similar results were obtained using TCGA GBM microarray data (including a higher number of GBM patients with both gene expression and DNA methylation information; *n* = 117; *r* = −0.25, *P* < 0.01; *r* = 0.32, *P* < 0.001, for Region 1 and Region 2, respectively; and for 5 of the individual probes; Fig. S3). Interestingly, looking for the 28 DNA methylation sites within the *WNT6* locus, in 516 LGG and 141 GBM patients, we identified regions that are consistently hypomethylated (e.g., from the 4th probe [cg16256504] to the 8th probe [cg02175741]) or hypermethylated (e.g., 16th probe [cg05618201]) both in LGG and in GBM (Figs 2A and S2), showing a remarkable homogeneity of DNA methylation levels of these particular regions across very heterogeneous glioma samples of different grades.

Having characterized *WNT6* DNA methylation levels in the large cohort from TCGA based on methylation arrays, we further characterized *WNT6* DNA methylation levels using MSP (Fig. S4) in an independent cohort of GBM samples (HSA, Portugal, *n* = 18). Primers for MSP were designed to detect the CpG’s recognized by the first two probes of selected Region 1 (Figs 2B and S1, and Table S1; cg11175192 and cg06157334). As observed in the methylation data from TCGA, a great percentage of GBM patients (55.6%) did not have detectable levels of *WNT6* DNA methylation in this region, as assessed by MSP (Fig. S4).

To further elucidate the role of *WNT6* DNA methylation in its expression, a panel of seven glioma cell lines was treated with 5‐Aza, a global DNA‐demethylating agent (Fig. 2D,E). MSP analyses showed that five of the seven cell lines presented 5‐Aza‐mediated demethylation (A172, SNB19, KNS42, SW1783, and Res186; Fig. 2D). Interestingly, 5‐Aza treatment successfully increased *WNT6* expression in four of these five cell lines (fold changes between 1.7 and 3.15; for KNS42, SW1783, A172, and Res186). Concordantly, the two cell lines in which MSP did not show an efficient demethylation of the *WNT6* promoter did not show increased *WNT6* expression (fold changes of 1 and 1.3 for UW479 and U87 cells, respectively; Fig. 2E). Together, these results suggest that *WNT6* DNA methylation levels contribute, at least partially, to regulate *WNT6* expression in glioma. Notwithstanding, other potential transcriptional regulatory mechanisms, such as *WNT6* promoter‐binding transcription factors, are likely to be involved in WNT6 activation in glioma.

### 3.3 *WNT6* is transcriptionally activated by HOXA9 in GBM

**Figure 3:**
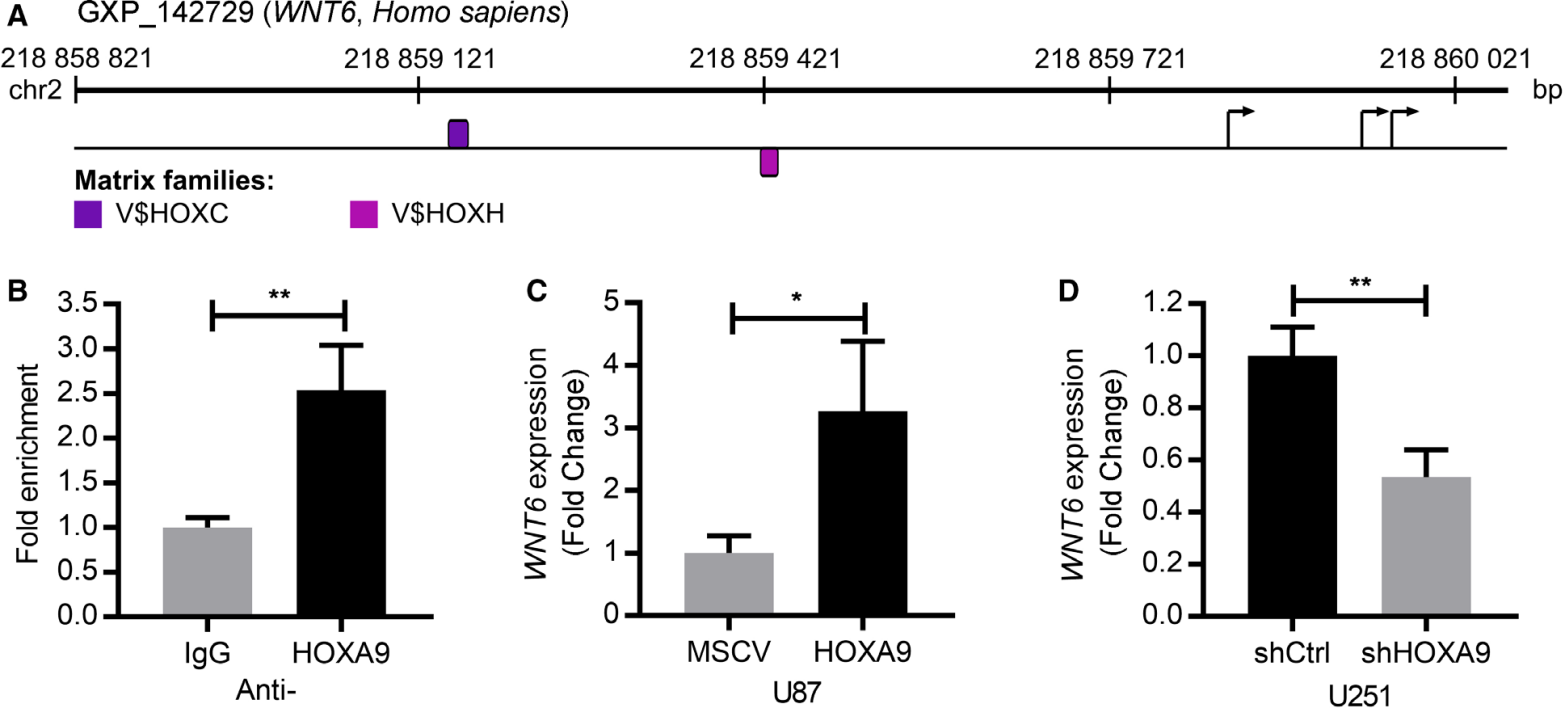
*WNT6* is a direct target of HOXA9 in GBM. (A) MatInspector representation of specific potential HOXA9 binding sites within *WNT6* promoter region. Violet and pink represent the matrices HOX_PBX.01 (matrix sim = 0.83, sequence: ggtgG*GAT*gg*ct*ggggg and family HOXC) and MEIS1A_HOXA9.01 (matrix sim = 0.86, sequence: *TGACag*g*tt*tgtt*g*a and family HOXH), respectively. Base pairs in italic appear in a position with a high conservation profile in the matrix (Ci‐value > 60). Base pairs in capital letters represent the core sequence used by the program. Matches represented on top of the sequence line were found on the positive strand, while matches found on the negative strand reside below the sequence line. Putative transcription start sites are marked by an arrow. Color codes for the matrix families are depicted below. (B) ChIP was performed to assess the putative binding of HOXA9 to the promoter region of *WNT6*, followed by qPCR. The fold enrichment presented is normalized to the input (DNA not exposed to immunoprecipitation—PCR‐positive control) and to the IgG background signal (ChIP‐negative control). Three independent experiments (mean and standard deviation) are represented. (C, D) *WNT6* qRT‐PCR was performed in U87 cells transfected to overexpress HOXA9 (U87‐HOXA9) and its negative counterparts (U87‐MSCV; C) and in U251‐shCtrl (HOXA9‐high) and U251‐shHOXA9 (HOXA9‐low) cells (D). Three independent experiments (mean and standard deviation) are represented. *P < 0.05 and **P < 0.01.

It was previously shown that *WNT6* expression is positively regulated by CAV1 (a scaffolding protein), *UCA1* (a long noncoding RNA), and PLAGL2 (a zinc finger protein) in gastric, bladder, and colorectal cancer, respectively (Fan *et al.*, 2014; Li *et al.*, 2019; Yuan *et al.*, 2013). However, while these genes have been described to be expressed in glioma (Abulrob *et al.*, 2004; Zhao *et al.*, 2017; Zheng *et al.*, 2010), we found the transcriptional levels of *WNT6* are not significantly correlated with *CAV1* (*r* = −0.03, *P* = 0.66) or *PLAGL2* (*r* = 0.07, *P* = 0.36) in human GBM, and *UCA1* and *WNT6* are inversely correlated (*r* = −0.29, *P* < 0.001). Thus, these molecules are unlikely responsible for promoting *WNT6* expression in GBM. Searching for other potential transcriptional regulators, we performed *in silico* analyses using the MatInspector tool (Genomatix) that locates putative bindings sites (based on matrix matches in DNA sequences) for multiple transcription factors. This revealed a significant number of potential transcription factors for *WNT6* (Fig. S5). Among them, we found 12 potential binding sites for homeobox proteins, including two for HOXA9 (Fig. 3A), which, interestingly, we previously showed to present various oncogenic roles and to be a prognostic biomarker in GBM patients (Costa *et al.*, 2010; Pojo *et al.*, 2015). Interestingly, a putative link between HOXA9 and *WNT6* has never been described. To test whether HOXA9 may effectively bind to the promoter region of *WNT6*, directly or as part of a larger protein complex, we performed anti‐HOXA9 ChIP assays on U251 GBM cells (endogenously expressing *HOXA9*) (Figs 3B and S1). We found that HOXA9 binds to *WNT6* promoter region in GBM cells (*P* < 0.01; Fig. 3B), therefore demonstrating that *WNT6* is a target of HOXA9 in GBM.

To test whether HOXA9 binding to the promoter region of *WNT6* activates its expression, we performed *WNT6* qRT‐PCR in GBM cell models engineered to overexpress or silence HOXA9 expression (Costa *et al.*, 2010; Pojo *et al.*, 2015). Interestingly, *WNT6* expression was significantly increased upon *HOXA9* overexpression in U87 cells (Fig. 3C) and decreased upon *HOXA9* silencing in U251 cells (Fig. 3D), strongly suggesting that HOXA9 binding to the *WNT6* promoter region promotes *WNT6* expression in GBM.

### 3.4 *WNT6* and *HOXA9* are co‐expressed in glioma patients

**Figure 4:**
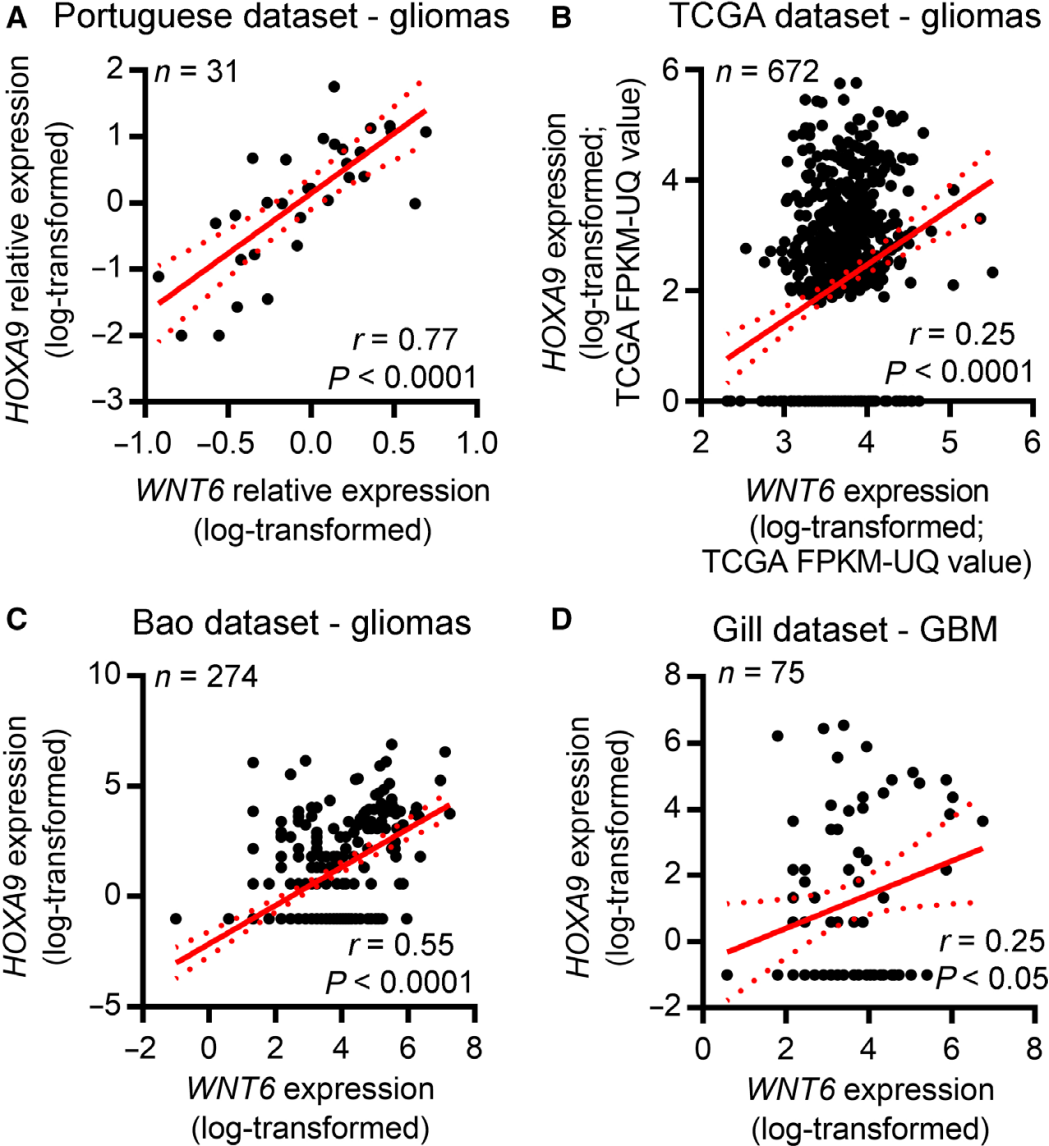
*WNT6* and *HOXA9* are co‐expressed in glioma patients. Correlation graphs between *WNT6* and *HOXA9* expression in glioma patients from (A) our Portuguese cohort (qRT‐PCR data; *n* = 31; *r* = 0.77, *P* < 0.0001), (B) the TCGA dataset (RNAseq data – log_10_‐transformed after adding a pseudocount of 1; *n* = 672; *r* = 0.25, *P* < 0.0001), (C) Bao dataset (GlioVis RNAseq data; *n* = 274; *r* = 0.55, *P* < 0.0001), and (D) Gill dataset (GlioVis RNAseq data; *n* = 75; *r* = 0.25, *P* < 0.05). The red line represents the linear regression line, while dashed curved lines represent 95% confidence intervals (CI).

To validate the association of *WNT6* and *HOXA9* in the clinical setting, the expression of these two genes was analyzed by qRT‐PCR in our cohort of glioma patients from HB, Portugal (*n* = 31; Fig. 4A), and by RNAseq data in three additional larger independent cohorts (TCGA, *n* = 672; Bao, *n* = 274; and Gill, *n* = 75; Fig. 4B–D). In all cohorts, *WNT6* and *HOXA9* levels were highly correlated (*r* = 0.77, *P* < 0.0001; *r* = 0.25, *P* < 0.0001; *r* = 0.55, *P* < 0.0001; *r* = 0.25, *P* < 0.05, for the Portuguese, TCGA, Bao, and Gill datasets, respectively).

**Figure 5:**
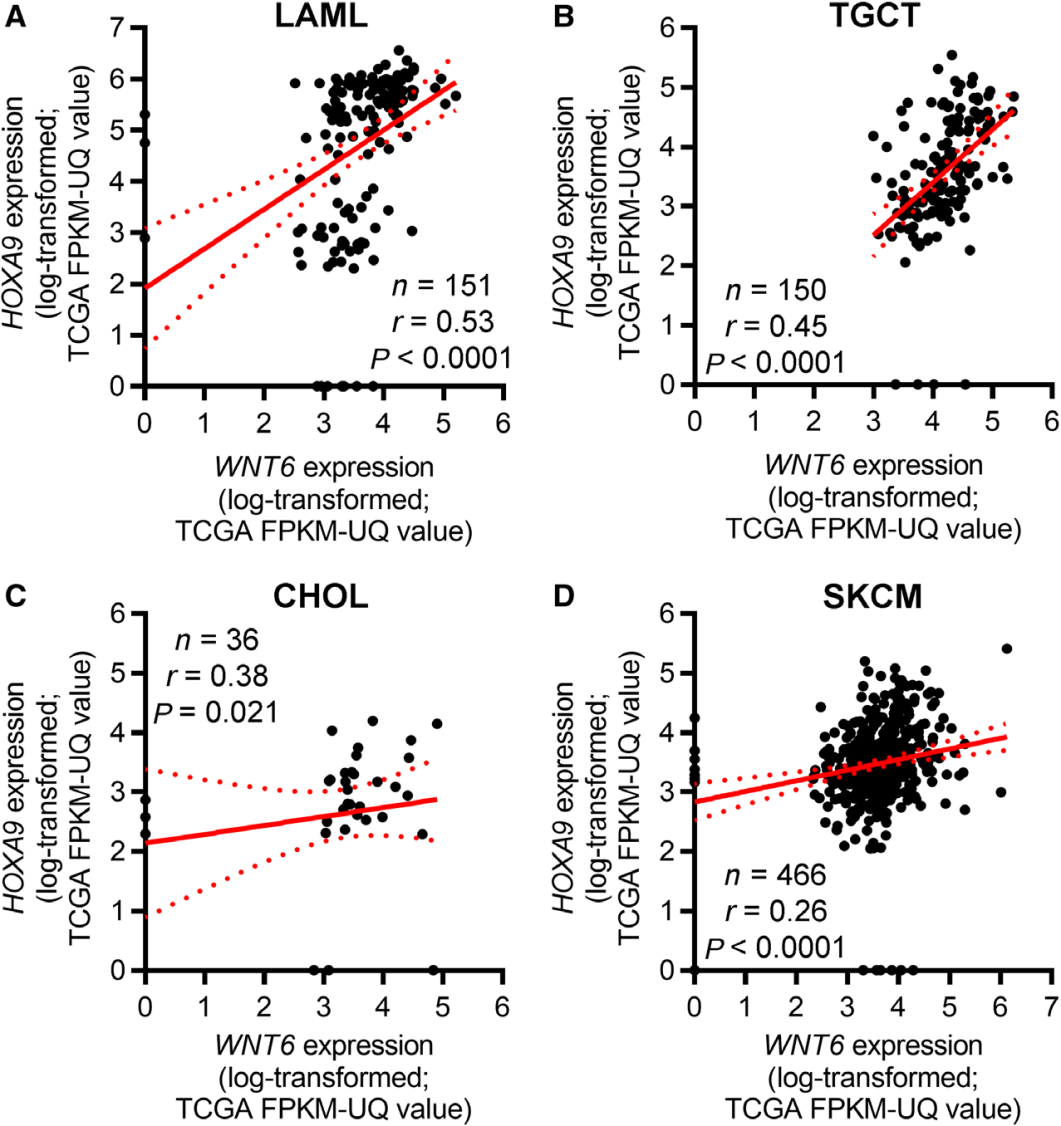
*WNT6* and *HOXA9* co‐expression is not exclusive of glioma tumors. Correlation graphs between *WNT6* and *HOXA9* expression in (A) acute myeloid leukemia (LAML; *n* = 151; *r* = 0.53, *P* < 0.0001); (B) testicular germ cell tumor (TGCT; *n* = 150; *r* = 0.45, *P* < 0.0001); (C) CHOL (*n* = 36; *r* = 0.38, *P* = 0.021); and (D) SKCM (*n* = 466; *r* = 0.26, *P* < 0.0001). The RNAseq data were log_10_‐transformed after adding a pseudocount of 1. The red line represents the linear regression line, while dashed curved lines represent 95% CI.

To understand whether this association is exclusive of glioma tumors, similar analyses were performed in all cancer types from TCGA with available RNAseq expression data. Interestingly, *WNT6* and *HOXA9* were also found to be co‐expressed in other cancer types, including leukemia, testicular germ cell tumor, melanoma, and CHOL (Table 1 and Fig. 5). Interestingly, when performing GSEA to identify transcriptomic signatures reminiscent of *WNT6*‐associated genes in GBM patients (Gonçalves *et al.*, 2018), we found that *WNT6*‐negatively correlated genes were enriched for genes upregulated in LAML cells upon HOXA9 knockdown [enrichment score (ES) = −0.26 and false discovery rate, FDR = 0.18; Fig. S6], further supporting this novel molecular link between HOXA9 and WNT6.

**Table 1:** *WNT6* and *HOXA9* co‐expression in all cancer types with available RNAseq data in TCGA. Bold‐faced values indicate strong correlations (−0.25 < *r* > 0.25) and significant *P*‐values.

Cancer code	Cancer designation	Number	*r* value	*P*‐value
ACC	Adrenocortical carcinoma	79	0.20	0.077
BLCA	Bladder urothelial carcinoma	408	−0.11	**0.029**
BRCA	Breast invasive carcinoma	1091	0.21	**< 0.0001**
CESC	Cervical squamous cell carcinoma and endocervical adenocarcinoma	304	0.00	0.989
CHOL	Cholangiocarcinoma	36	**0.38**	**0.021**
COAD	Colon adenocarcinoma	456	0.05	0.313
DLBC	Lymphoid neoplasm diffuse large B‐cell lymphoma	48	0.02	0.892
ESCA	Esophageal carcinoma	161	−0.07	0.367
HNSC	Head and neck squamous cell carcinoma	500	0.07	0.108
KICH	Kidney chromophobe	65	0.07	0.596
KIRC	Kidney renal clear cell carcinoma	530	−0.01	0.845
KIRP	Kidney renal papillary cell carcinoma	288	−0.10	0.088
LAML	Acute myeloid leukemia	151	**0.53**	**< 0.0001**
LIHC	Liver hepatocellular carcinoma	371	0.09	0.061
LUAD	Lung adenocarcinoma	513	0.23	**< 0.0001**
LUSC	Lung squamous cell carcinoma	501	0.17	**0.0001**
MESO	Mesothelioma	86	0.20	0.058
PAAD	Pancreatic adenocarcinoma	177	0.15	**0.044**
PCPG	Pheochromocytoma and paraganglioma	179	0.04	0.579
PRAD	Prostate adenocarcinoma	495	−0.07	0.104
READ	Rectum adenocarcinoma	166	0.07	0.359
SARC	Sarcoma	259	−0.05	0.416
SKCM	Skin cutaneous melanoma	466	**0.26**	**< 0.0001**
STAD	Stomach adenocarcinoma	375	−0.07	0.172
TGCT	Testicular germ cell tumors	150	**0.45**	**< 0.0001**
THCA	Thyroid carcinoma	502	0.17	**0.0001**
THYM	Thymoma	119	−0.22	**0.018**
UCS	Uterine carcinosarcoma	56	−0.22	0.108
UVM	Uveal melanoma	80	0.04	0.718

### 3.5 HOXA9 activates the WNT/β‐catenin pathway

**Figure 6:**
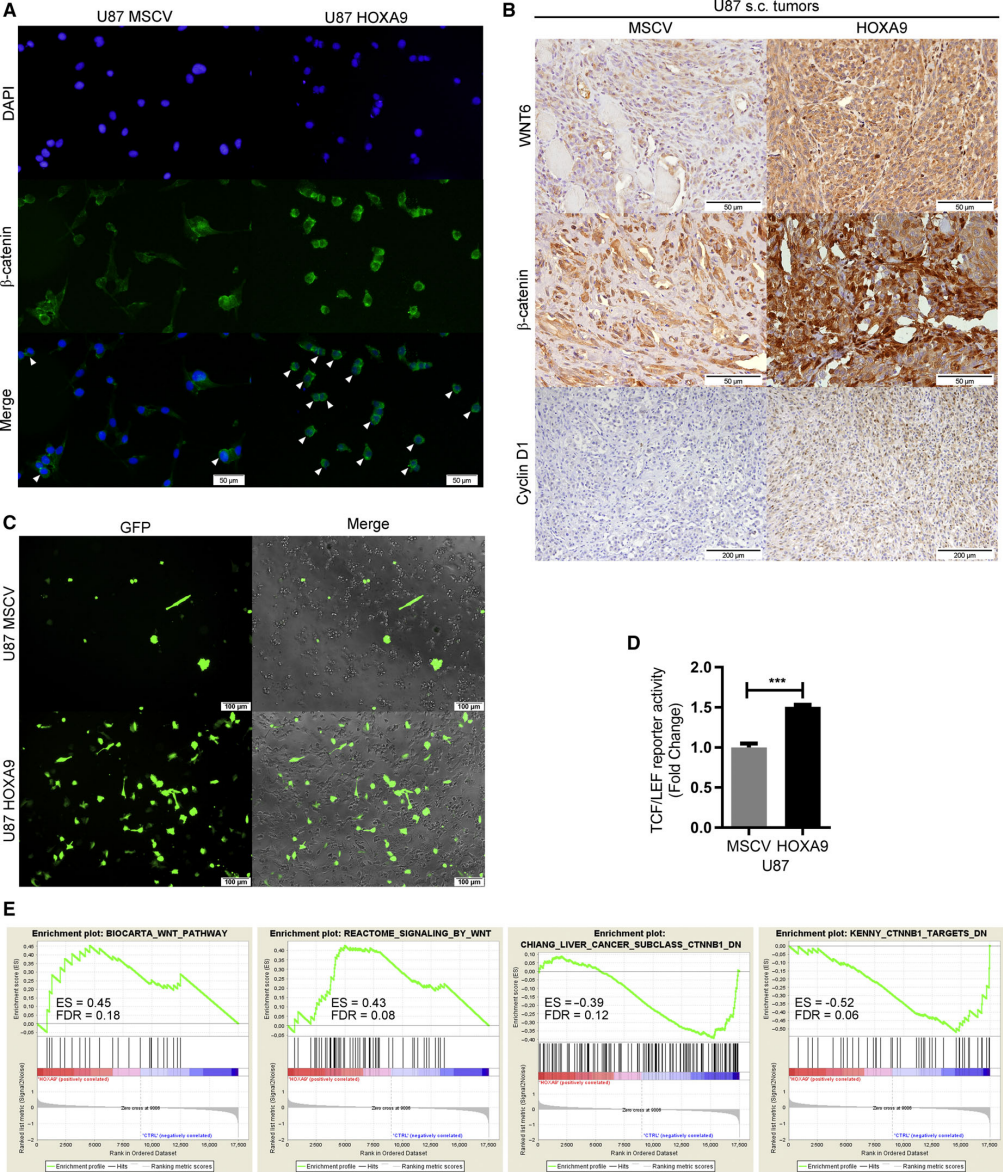
HOXA9 activates the WNT/β‐catenin pathway in *in vitro* and *in vivo* models of GBM. (A) β‐catenin expression and subcellular localization in HOXA9‐negative (U87‐MSCV) and HOXA9‐positive (U87‐HOXA9) GBM cells were evaluated by IF. White arrowheads indicate perinuclear stained cells. Representative images are displayed (200× magnification; scale bar = 50 µm). (B) WNT6, β‐catenin, and cyclin D1 expression was analyzed by IHC in subcutaneous tumors formed upon injection of HOXA9‐negative and HOXA9‐positive U87 GBM cells in immunocompromised mice. Representative images are displayed (scale bars are specified in each image). (C, D) TCF/LEF reporter assay in U87‐MSCV (HOXA9‐negative) and U87‐HOXA9. (C) Representative images are displayed (100× magnification; scale bar = 100 µm). Merged images represent the combination of GFP and contrast‐phase photographs. (D) GFP expression was used as a measure of TCF/LEF promoter activity and was normalized against negative and positive controls. (*n* = 3 independent assays; mean ± SD; ***P < 0.001, two‐sided unpaired *t*‐test). (E) GSEA querying the transcriptome of HOXA9 in U87 cells (Pojo et al., 2015), highlighting gene expression signatures associated with WNT signaling.

We previously described that WNT6 is an activator of the WNT/β‐catenin pathway in GBM (Gonçalves *et al.*, 2018). Thus, we aimed to understand whether HOXA9, being a transcriptional activator of WNT6, may also increase the activity of this pathway. By performing β‐catenin IF in U87‐HOXA9 cells and their HOXA9‐negative counterparts (MSCV; Fig. 6A), we found β‐catenin protein expression was significantly increased in U87‐HOXA9 cells, staining both the cytoplasm and the nucleus. This association was not only observed *in vitro* but also *in vivo*, as U87+/−HOXA9 tumors grown subcutaneously in nude mice also showed significantly higher expression of WNT6 and β‐catenin (mainly in the nucleus) in HOXA9‐positive tumors when compared to HOXA9‐negative tumors (Fig. 6B). In addition, cyclin D1, a known transcriptional target of the canonical WNT/β‐catenin pathway, was also upregulated in HOXA9‐positive tumors when compared to negative tumors (Fig. 6B).

To further confirm that HOXA9 can activate the canonical WNT pathway, TCF/LEF reporter assays were performed in U87‐MSCV and U87‐HOXA9 cells. In accordance with the above results, a significantly increased activation of the WNT canonical pathway was observed in HOXA9‐high GBM cells when compared to their negative counterparts (*P* < 0.001; Fig. 6C,D).

Interestingly, querying a previously generated HOXA9‐associated transcriptomic signature (Pojo *et al.*, 2015) through GSEA revealed that HOXA9‐deregulated genes in U87MG cells are significantly associated with genes involved in the WNT pathway in four independent datasets (ES = 0.45 and false discovery rate, FDR = 0.18; ES = 0.43 and FDR = 0.08; ES = −0.39 and FDR = 0.12; and ES = −0.52 and FDR = 0.06; Fig. 6E), globally suggesting that the transcriptome of HOXA9 is associated with WNT signaling.

Together, these results, integrating *in vitro* and *in vivo* data, point out HOXA9 as a transcriptional activator of *WNT6*, consequently activating the canonical WNT (β‐catenin‐dependent) pathway in GBM.

### 3.6 *WNT6* is prognostically valuable independently of *HOXA9* expression in GBM patients

**Figure 7:**
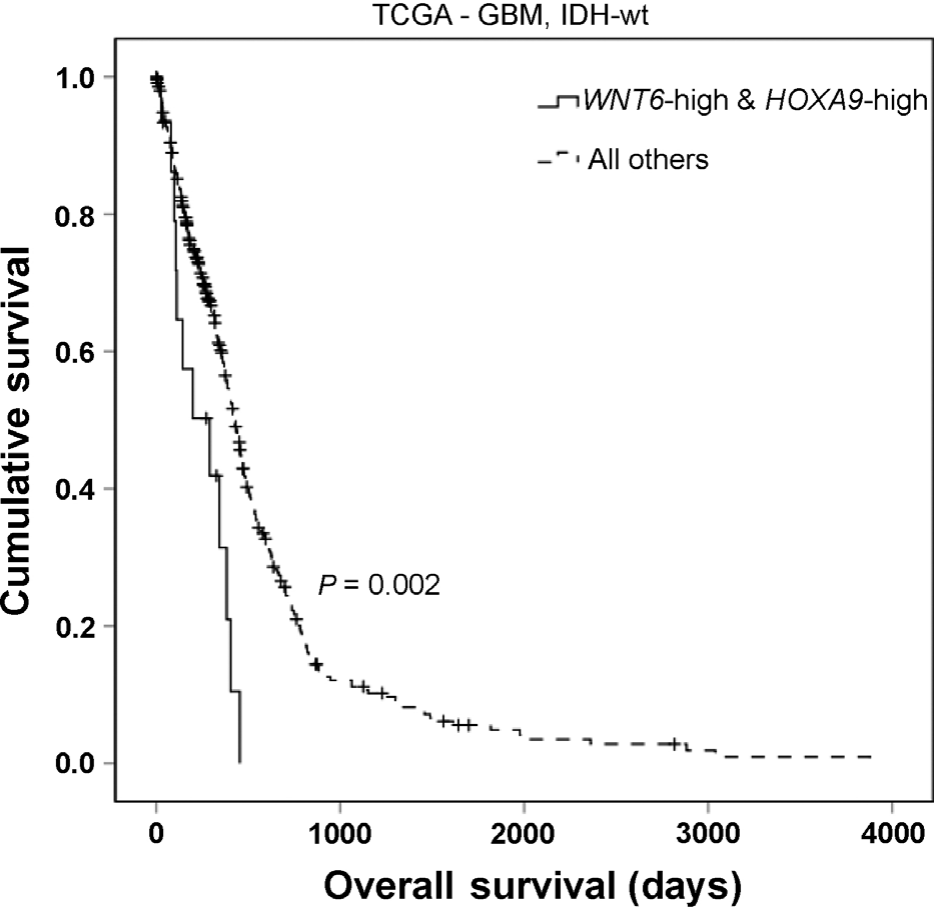
Concomitant high *WNT6* and *HOXA9* expression identifies a subgroup of IDHwt GBM patients with particular dismal prognosis. Kaplan–Meier OS curve of *WNT6*‐high and *HOXA9*‐high patients (median OS = 290 days) *vs*. all others (median OS = 425 days) in IDHwt GBM patients (*n* = 367; *P* = 0.002, log‐rank test).

We previously described that WNT6 (Gonçalves *et al.*, 2018) and *HOXA9* (Costa *et al.*, 2010; Pojo *et al.*, 2015) are each associated with decreased overall survival (OS) of GBM patients. In this work, we also investigated whether they would maintain their clinical significance independently of each other. Thus, the clinical impact of *WNT6* in GBM was evaluated using a multivariable Cox model to adjust to potential confounding effects of other putative prognostic factors, namely patient age, KPS, gender, therapy, IDH mutation status, and *HOXA9* expression (Tables 2 and S2). Interestingly, *WNT6* expression is associated with shorter OS of GBM patients, independently of *HOXA9* expression and all other putative prognostic variables in GBM patients from the TCGA (*n* = 293; *P* = 0.012), with *HOXA9* also maintaining its clinical significance (*P* = 0.002; Table 2). Importantly, IDHwt GBM patients with both *WNT6*‐high and *HOXA9*‐high expression presented a shorter OS (median OS = 290 days) when compared to all other patients (median OS = 425; log‐rank *P* = 0.002; Fig. 7). Moreover, high expression of *WNT6* allowed the identification of a subgroup of HOXA9‐low GBM patients with a significantly shorter OS (median OS = 298 days) than those with low *WNT6* levels (median OS = 447 days; log‐rank *P* = 0.013; Fig. S7). These results suggest that both *WNT6* and *HOXA9* are critical and informative prognostic biomarkers in GBM patients.

**Table 2:** Cox multivariable survival analysis in GBM patients from TCGA. *n* = 293—microarray data. Bold‐faced values indicate significant *P*‐values.

	OS		
	*P*‐value	Hazard ratio	95% CI
*WNT6* expression^a^	**0.012**	1.448	1.09–1.93
Age at diagnosis^a^	**<0.0001**	1.026	1.01–1.04
KPS^a^	**0.002**	0,983	0.97–0.99
Gender^b^	**0.046**	0.737	0.55–0.99
Treatment^c^	**< 0.0001**	0.196	0.12–0.34
IDH1 status^d^	0.129	0.635	0.35–1.14
*HOXA9* expression^e^	**0.002**	2.505	1.40–4.48

a *WNT6* expression, age, and KPS were used as continuous variables

b Female *vs.* male

c Nontreated *vs.* treated

d IDHwt vs IDHmut

e *HOXA9*‐low *vs. HOXA9*‐high expression.

## 4 Discussion

WNT ligands are morphogen molecules important during embryogenesis (Nusse and Clevers, 2017; Willert and Nusse, 2012), whose deregulated expression has been described in cancer, including GBM (Binda *et al.*, 2017; Hu *et al.*, 2016; Kamino *et al.*, 2011; Kaur *et al.*, 2013; Kim *et al.*, 2015b; Pu *et al.*, 2009; Yu *et al.*, 2007). We recently showed that the WNT6 ligand is overexpressed in GBM and is associated with tumor aggressiveness *in vitro* and *in vivo* (Gonçalves *et al.*, 2018). However, the mechanisms underlying WNT6 overexpression in GBM were still unknown. In the present study, we investigated the upstream mechanisms regulating WNT6 in GBM, analyzing *WNT6* copy number alterations, DNA methylation, and its link to putative direct transcriptional regulators.

Our data showed that *WNT6*‐high expression in glioma increases with grade independently of IDH mutation and 1p/19q codeletion status (Fig. 1). This result suggests that *WNT6* expression associates with glioma increased malignancy independently of the remarkable differences between glioma molecular subtypes (astrocytoma IDHwt, astrocytoma IDHmut, and IDHmut 1p/19q codeleted oligodendroglioma). Considering this result, in the future it will be interesting to investigate the potential value of WNT6 for patient stratification. At the light of previous findings regarding the relevance of WNT6 promoting resistance to chemotherapy (Gonçalves *et al.*, 2018), it will also be interesting to understand whether WNT6 may be a clinically useful biomarker predictive of therapy response, similarly to what has been done for *MGMT* promoter methylation status within IDHwt gliomas, in which patients whose tumors lack *MGMT* promoter methylation are treated with radiotherapy only (Louis *et al.*, 2016).

To reach our goal of understanding the mechanisms underlying *WNT6* activation in glioma, we integrated data from (epi)genetic and *in silico* analyses from patients and cell lines. First, we observed that *WNT6* is expressed in a gene dosage‐independent manner in glioma (Fig. 1). In contrast, our findings demonstrated that DNA methylation, a critical epigenetic mechanism, associates with *WNT6* expression levels in glioma (Figs 2, S2 and S3), similarly to what was observed for other WNT ligands in other cancer types (Carmona *et al.*, 2013; Jung *et al.*, 2015; Kim *et al.*, 2015a; Liu *et al.*, 2016; Xu *et al.*, 2005). In particular, we observed that higher levels of DNA methylation, in particular CpGs of the promoter region, are associated with *WNT6* silencing, while gene body methylation is positively associated with its expression (Figs 2, S2 and S3). This is in agreement with the known effects of promoter and intragenic DNA methylation in gene expression regulation in normal and cancer cells (Kulis *et al.*, 2012; Lim *et al.*, 2017; Long *et al.*, 2017; Yang *et al.*, 2014). Interestingly, most of the CpG sites are more frequently methylated in LGG than GBM patients (19 out of 28; Fig. 2), suggesting that *WNT6* locus may be globally hypomethylated during tumor progression.

Although DNA methylation was clearly associated with *WNT6* expression in glioma, this association was not universal. After ruling out the potential regulation of *WNT6* by CAV1, *UCA1*, and PLAGL2 in GBM (contrasting to what was previously described in gastric, bladder, and colorectal cancers (Fan *et al.*, 2014; Li *et al.*, 2019; Yuan *et al.*, 2013)), we showed that HOXA9, an oncogenic transcription factor whose expression has been associated with increased GBM aggressiveness (impacting on several hallmarks of cancer, increasing cell viability, migration, resistance to TMZ, and tumor growth in a subcutaneous mouse model, while decreasing mice OS in an orthotopic mouse model) and patients’ poor survival (Costa *et al.*, 2010; Pojo *et al.*, 2015), is one of the *WNT6* transcriptional regulators in GBM (Fig. 3), binding to its promoter region. Concordantly, *HOXA9* expression correlates significantly with *WNT6* in GBM patients and cell models. These observations further strengthen our data suggesting an important role of HOXA9 for *WNT6* transcriptional activation. Notwithstanding, further studies are warranted, as other transcription factors, as well as other regulatory mechanisms, might also be implicated in the regulation of *WNT6* in glioma. Moreover, *WNT6* and *HOXA9* were not only co‐expressed in glioma (Fig. 4), but also in leukemia, a cancer type where the oncogenic roles of HOXA9 are well established (Armstrong *et al.*, 2002; Esposito *et al.*, 2015; Kroon *et al.*, 2001; Lawrence *et al.*, 1999), and also in TGCT, CHOL, and melanoma (Fig. 5 and Table 1). Thus, it would be interesting to evaluate whether HOXA9 also activates *WNT6* expression in LAML and whether WNT6 also displays oncogenic roles in those malignancies.

We also demonstrated that WNT6 and HOXA9 cooperate in activating the WNT/β‐catenin signaling pathway in GBM (Fig. 6), suggesting WNT6 and the WNT/β‐catenin signaling pathway as effectors of HOXA9‐mediated aggressiveness in GBM (Costa *et al.*, 2010; Pojo *et al.*, 2015). Interestingly, it was recently reported that HOXA genes are part of a gene‐signature characteristic of WNT‐dependent glioma stem cells (Rajakulendran *et al.*, 2019), which fits well with our data linking HOXA9 and WNT6 in GBM. This link is particularly relevant from a clinical perspective, as it may reveal novel therapeutic opportunities targeting WNT signaling to revert the malignant behaviors of highly aggressive WNT6‐high and HOXA9‐high GBMs, all of which present particularly poor clinical outcome. Importantly, we showed that *WNT6* and *HOXA9* are prognostically valuable, independently of each other, in GBM patients, and that their concomitant high expression identifies a subgroup of patients with very dismal prognosis (Table 2, Figs 7 and S7). These findings support the clinical evaluation of these two biomarkers and suggest that WNT6 may also have oncogenic roles and mechanisms of transcriptional regulation beyond those dependent on HOXA9. Interestingly, WNT6 was also shown to be associated with shorter survival in LGG patients (Dao Trong *et al.*, 2018), where *HOXA9* overexpression is not frequent (Pojo *et al.*, 2015). Future works should explore the potential therapeutic value of targeting the HOXA9/WNT6/WNT pathway axis in GBM, for which novel treatments are urgently needed. Moreover, it will be interesting to understand whether WNT6 may influence patient outcome by modulating the tumor microenvironment/immunologic landscape (Pai *et al.*, 2017), or even by regulating the glioma stem cells population (Gonçalves *et al.*, 2018), thus influencing tumor pathophysiology/recurrence and ultimately patient prognosis.

## 5 Conclusions

This work provides significant insights on the (epi)genetic mechanisms underlying WNT6 activation in highly aggressive GBMs, which may potentially influence GBM patients’ management by aiding treatment decisions and prognostic stratifications, while also opening new opportunities to identify or develop potentially more effective therapies for these highly resistant tumors.

## Conflict of interest

The authors declare no conflict of interest.

## Author contributions

CSG, NS, and BMC designed research studies. CSG, AXM, and EPM conducted experiments. CSG, AXM, EPM, NS, and BMC analyzed data. AAP, MMP, CP, and RMR provided patient samples. CSG and BMC wrote the manuscript, which was reviewed by all authors.

## Supporting information


**Table S1.** Probes from Illumina Infinium DNA methylation 450 array used to query methylation levels at the *WNT6* gene locus.
**Table S2.** Cox multivariable survival analysis in GBM patients from TCGA.
**Fig. S1.** Schematic representation of the *WNT6* locus, showing the localization of MSP and ChIP PCR products, and their relative localization to Region 1 (from Figure 2) and to HOXA9 potential binding sites (identified in Figure 3).
**Fig. S2.** *WNT6 *DNA methylation correlates with *WNT6* expression in gliomas.
**Fig. S3.** Validation of the regulation of *WNT6 *transcription by DNA methylation in gliomas, using TCGA microarray data.
**Fig. S4.** *WNT6 *DNA methylation in a Portuguese GBM cohort.
**Fig. S5.** WNT6 potential transcription factors.
**Fig. S6.** *WNT6*‐correlated genes enriched for HOXA9 target genes.
**Fig. S7.** *WNT6* expression identifies a subgroup of patients with shorter OS in *HOXA9*‐low IDH‐wildtype GBM patients.
