# Supplementary material for: A novel molecular link between HOXA9 and WNT6 in glioblastoma identifies a subgroup of patients with particular poor prognosis
Source: Mol Oncol. 2020 May 6;14(6):1224–41. doi: 10.1002/1878-0261.12633 (PMC7266278; doi:10.1002/1878-0261.12633)
Supplement: Supplementary file 1 — Table S1. Probes from Illumina Infinium DNA methylation 450 array used to query methylation levels at the WNT6 gene locus. Table S2. Cox multivariable survival analysis in GBM patients from TCGA. Fig. S1. Schematic representation of the WNT6 locus, showing the localization of MSP and ChIP PCR products, and their relative localization to Region 1 (from Figure 2) and to HOXA9 potential binding sites (identified in Figure 3). Fig. S2. WNT6 DNA methylation correlates with WNT6 expression in gliomas. Fig. S3. Validation of the regulation of WNT6 transcription by DNA methylation in gliomas, using TCGA microarray data. Fig. S4. WNT6 DNA methylation in a Portuguese GBM cohort. Fig. S5. WNT6 potential transcription factors. Fig. S6. WNT6‐correlated genes enriched for HOXA9 target genes. Fig. S7. WNT6 expression identifies a subgroup of patients with shorter OS in HOXA9‐low IDH‐wildtype GBM patients. [file MOL2-14-1224-s001.pdf]

Gonçalves *et al.*, “A novel molecular link between HOXA9 and WNT6 in glioblastoma identifies a subgroup of patients with particular poor prognosis”

Supplementary Data, including:

- **Supplementary Tables;**
- **Supplementary Figure Legends;**
- **Supplementary Figures.**

Supplementary Tables

Supplementary Table 1. Probes from Illumina Infinium DNA methylation 450 array used to query methylation levels at the *WNT6* gene locus

Number	Probe	Location on chr2	Details	Selected regions (Fig 2 and Sup Fig 2)
1	cg17511968	218856442		
2	cg00037013	218857813		
3	cg05985303	218858982	CpG island on 5' promoter region - exon 1	
4	cg16256504	218859434		
5	cg10000424	218859681		
6	cg07714657	218859685		
7	cg06157334	218859816		
8	cg02175741	218860168		Region 1
9	cg11175192	218860195		
10	cg22156632	218860346		
11	cg15074329	218860746		
12	cg05420495	218860984		
13	cg11511777	218861236		
14	cg14420663	218863242		
15	cg26302230	218869117		
16	cg05618201	218869888		
17	cg00981994	218871154		
18	cg24813176	218871445	CpG island exon 3	
19	cg23154059	218871528		
20	cg22344703	218871590		
21	cg06862374	218871827		
22	cg06795233	218872670		
23	cg22587479	218873504	CpG island exon 4	Region 2
24	cg00011225	218873592		
25	cg14639163	218873807		
26	cg13903421	218873992		
27	cg25242471	218874010		
28	cg07653647	218876807		

Supplementary Table 2. Cox multivariable survival analysis in GBM patients from TCGA

	Overall Survival		
	***p*-value**	**HR**	**95% CI**
***WNT6* expression**[a]	**0.041**	1.27	1.01-1.60
**Age at diagnosis**[a]	**<0.0001**	1.03	1.02-1.04
**KPS**[a]	**0.001**	0.98	0.97-0.99
**Gender**[b]	**0.03**	0.76	0.60-0.97
**Treatment**[c]	**<0.0001**	0.19	0.12-0.30
***HOXA9* expression**[d]	**0.022**	1.82	1.09-3.04

[a]*WNT6* expression, age and KPS were used as continuous variables; [b]female vs. male; [c]non-treated vs. treated; [d]*HOXA9*-low vs. *HOXA9*-high expression.

n = 405 – microarray data; HR: Hazard ratio; CI: Confidence intervals

Supplementary Figure Legends

Figure S1. Schematic representation of the *WNT6* locus, showing the localization of MSP and ChIP PCR products, and their relative localization to Region 1 (from Figure 2) and to HOXA9 potential binding sites (identified in Figure 3). Putative transcription start sites (TSS) are marked by arrows. Color codes are depicted below, and chromosomal coordinates are indicated above.

Figure S2. *WNT6* DNA methylation correlates with *WNT6* expression in gliomas. Correlation graphs between *WNT6* expression (x-axis) and DNA methylation from different CpG sites (y-axis) in LGG **(A)** and GBM **(B)**. The indicated probe number is based on Figure 2 and Supplementary Table 1. Spearman's correlation test *r* values are indicated. *, $p < 0.05$; **, $p < 0.01$; ***, $p < 0.005$; and ****, $p < 0.001$.

Figure S3. Validation of the regulation of *WNT6* transcription by DNA methylation in gliomas, using TCGA microarray data.
(A) Heatmap representation of DNA methylation levels (TCGA β-values) corresponding to *WNT6* locus in 141 GBM (bottom) patients from TCGA microarray data. Each column corresponds to a probe and each row to a patient. A total of 28 methylation probes (vertical blue bars) were assessed. CpG islands >300 bp are represented in green. Coding exons are represented by blocks connected by lines representing introns. White rectangles at the left and right ends represent the 5' and 3' UTR, respectively. The methylation color code with TCGA β-values is shown on the left. Patients are ranked based on *WNT6* expression (obtained by microarray for 117/141 GBMs), as shown at the right. The percentage of highly methylated cases (TCGA β-values ≥ 0.5) for each CpG probe are depicted below the heatmap. Probes whose methylation levels were statistically correlated with *WNT6* expression levels ($|r| > 0.15$ and $p < 0.05$) are marked with * above the respective column (Spearman's correlation). **(B-C)** Correlation graphs between *WNT6* expression (TCGA level 3 value, microarray data) and average DNA methylation index (TCGA β-values) of the probes from both selected regions (Regions 1 and 2; B) and the individual statistically correlated probes (C) of 117 GBM. More details

about the probes are presented in Supplementary Table 1. Spearman's correlation test r values are indicated. *, $p < 0.05$; **, $p < 0.01$; and ***, $p < 0.005$.

Figure S4. *WNT6* DNA methylation in a Portuguese GBM cohort.

MSP analyses of 18 GBM patients from Hospital Santo António. A methylated case was considered whenever a clear band corresponding to the "methylated" reaction was observed and detected by the AzureSpot software (signal intensity values between 7718 and 16533 above the background). Top: representative image. Bottom: summary of the results.

Figure S5. WNT6 potential transcription factors.

MatInspector (Genomatix) representation of potential matrix families of transcription factors in the *WNT6* promoter region. Each matrix match is represented by a colored rectangle and each color represents a matrix family. Putative transcription start sites (TSS) are marked by an arrow. Color codes for the matrix families are depicted below.

Figure S6. WNT6-correlated genes enriched for HOXA9 target genes.

GSEA analysis showed that *WNT6*-negatively correlated genes in GBM patients (Gonçalves et al., 2018) were enriched for genes up-regulated in acute myeloid leukemia cells upon HOXA9 knockdown (ES = -0.26 and FDR = 0.18).

Figure S7. *WNT6* expression identifies a subgroup of patients with shorter OS in *HOXA9*-low IDH-wildtype GBM patients.

Kaplan-Meier overall survival curve of *WNT6*-high patients (median OS = 298 days) versus *WNT6*-low patients (median OS = 447 days) in *HOXA9*-low, IDH-wildtype GBM patients (n = 340; $p = 0.002$, Log-rank test; *WNT6*-high > 0.82 TCGA level 3 value).

Supplementary data references

Gonçalves, C.S., Vieira de Castro, J., Pojo, M., Martins, E.P., Queirós, S., Chautard, E., Taipa, R., Pires, M.M., Pinto, A.A., Pardal, F., Custódia, C., Faria, C.C., Clara, C., Reis, R.M., Sousa, N., Costa, B.M., 2018. WNT6 is a Novel Oncogenic Prognostic Biomarker in Human Glioblastoma. Theranostics 8, 4805-4823.

Figure S1

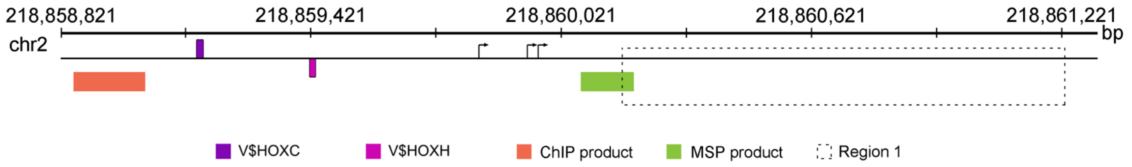
218,858,821
218,859,421
218,860,021
218,860,621
218,861,221
bp
chr2
V$HOXC
V$HOXH
ChIP product
MSP product
Region 1

Figure S2

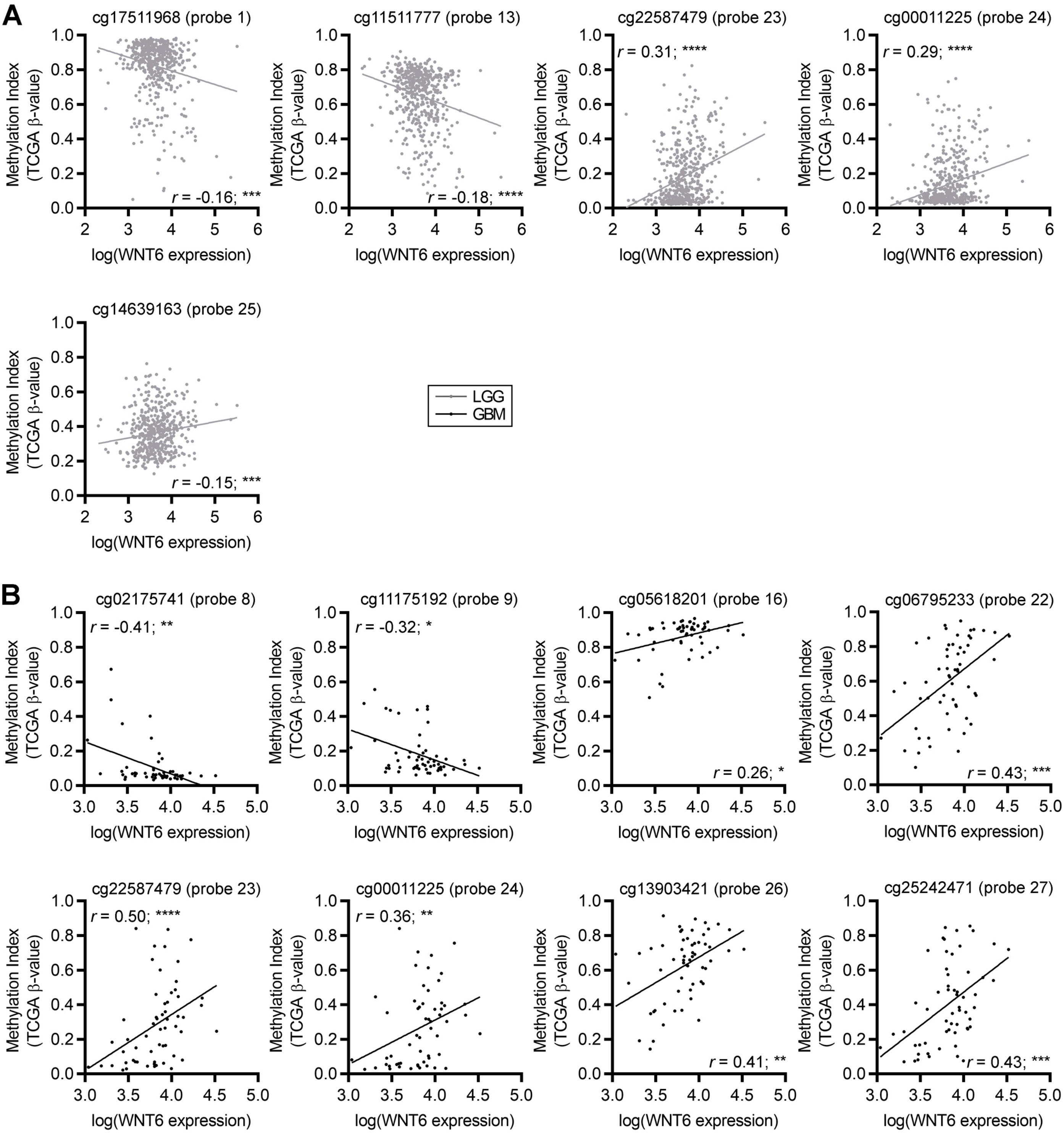

A
cg17511968 (probe 1)
r = -0.16; ***
cg11511777 (probe 13)
r = -0.18; ****
cg22587479 (probe 23)
r = 0.31; ****
cg00011225 (probe 24)
r = 0.29; ****
cg14639163 (probe 25)
r = -0.15; ***
LGG
GBM
B
cg02175741 (probe 8)
r = -0.41; **
cg11175192 (probe 9)
r = -0.32; *
cg05618201 (probe 16)
r = 0.26; *
cg06795233 (probe 22)
r = 0.43; ***
cg22587479 (probe 23)
r = 0.50; ****
cg00011225 (probe 24)
r = 0.36; **
cg13903421 (probe 26)
r = 0.41; **
cg25242471 (probe 27)
r = 0.43; ***
Methylation Index (TCGA β-value)
log(WNT6 expression)

Figure S3

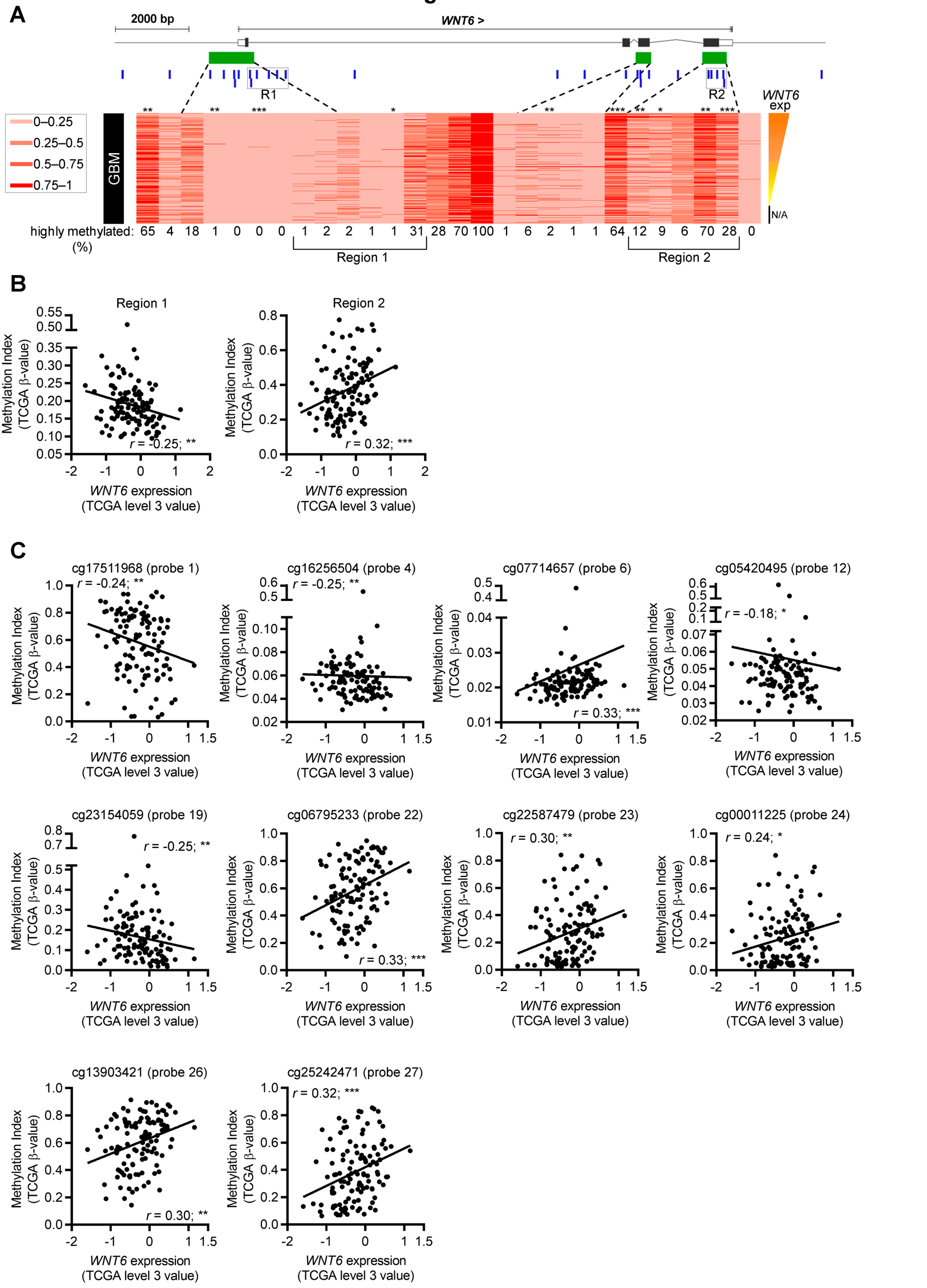
A
2000 bp
WNT6 >
R1
R2
WNT6 exp
0–0.25
0.25–0.5
0.5–0.75
0.75–1
GBM
N/A
highly methylated: (%)
65 4 18 1 0 0 0 1 2 2 1 1 31 28 70 100 1 6 2 1 1 64 12 9 6 70 28 0
Region 1
Region 2
B
Region 1
r = -0.25; **
Region 2
r = 0.32; ***
Methylation Index (TCGA β-value)
WNT6 expression (TCGA level 3 value)
C
cg17511968 (probe 1)
r = -0.24; **
cg16256504 (probe 4)
r = -0.25; **
cg07714657 (probe 6)
r = 0.33; ***
cg05420495 (probe 12)
r = -0.18; *
cg23154059 (probe 19)
r = -0.25; **
cg06795233 (probe 22)
r = 0.33; ***
cg22587479 (probe 23)
r = 0.30; **
cg00011225 (probe 24)
r = 0.24; *
cg13903421 (probe 26)
r = 0.30; **
cg25242471 (probe 27)
r = 0.32; ***

Figure S4

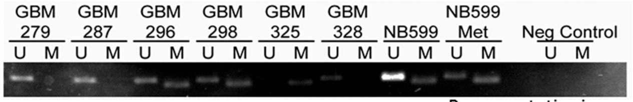

Representative image

WNT6 methylation status	Unmethylated	Methylated
Number of patients	10/18	8/18
Percentage	55.6%	44.4%

Figure S5

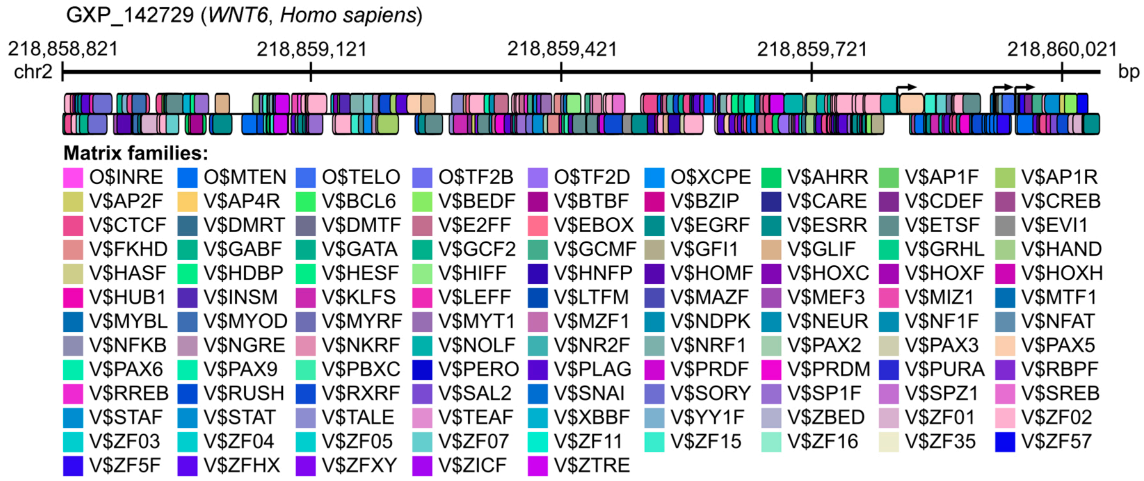
GXP_142729 (*WNT6*, *Homo sapiens*)
218,858,821
218,859,121
218,859,421
218,859,721
218,860,021
chr2
bp
Matrix families:
O$INRE
O$MTEN
O$TELO
O$TF2B
O$TF2D
O$XCPE
V$AHRR
V$AP1F
V$AP1R
V$AP2F
V$AP4R
V$BCL6
V$BEDF
V$BTBF
V$BZIP
V$CARE
V$CDEF
V$CREB
V$CTCF
V$DMRT
V$DMTF
V$E2FF
V$EBOX
V$EGRF
V$ESRR
V$ETSF
V$EVI1
V$FKHD
V$GABF
V$GATA
V$GCF2
V$GCMF
V$GFI1
V$GLIF
V$GRHL
V$HAND
V$HASF
V$HDBP
V$HESF
V$HIFF
V$HNFP
V$HOMF
V$HOXC
V$HOXF
V$HOXH
V$HUB1
V$INSM
V$KLFS
V$LEFF
V$LTFM
V$MAZF
V$MEF3
V$MIZ1
V$MTF1
V$MYBL
V$MYOD
V$MYRF
V$MYT1
V$MZF1
V$NDPK
V$NEUR
V$NF1F
V$NFAT
V$NFKB
V$NGRE
V$NKRF
V$NOLF
V$NR2F
V$NRF1
V$PAX2
V$PAX3
V$PAX5
V$PAX6
V$PAX9
V$PBXC
V$PERO
V$PLAG
V$PRDF
V$PRDM
V$PURA
V$RBPF
V$RREB
V$RUSH
V$RXRF
V$SAL2
V$SNAI
V$SORY
V$SP1F
V$SPZ1
V$SREB
V$STAF
V$STAT
V$TALE
V$TEAF
V$XBBF
V$YY1F
V$ZBED
V$ZF01
V$ZF02
V$ZF03
V$ZF04
V$ZF05
V$ZF07
V$ZF11
V$ZF15
V$ZF16
V$ZF35
V$ZF57
V$ZF5F
V$ZFHX
V$ZFXY
V$ZICF
V$ZTRE

Figure S6

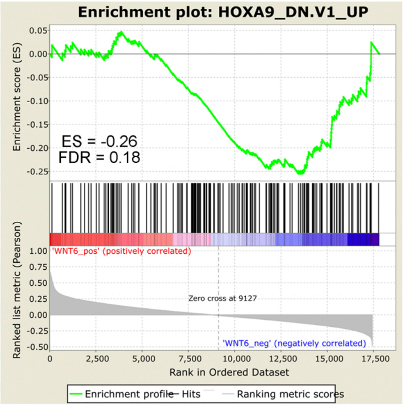
Enrichment plot: HOXA9_DN.V1_UP
Enrichment score (ES)
ES = -0.26
FDR = 0.18
Ranked list metric (Pearson)
'WNT6_pos' (positively correlated)
Zero cross at 9127
'WNT6_neg' (negatively correlated)
Rank in Ordered Dataset
Enrichment profile
Hits
Ranking metric scores

Figure S7

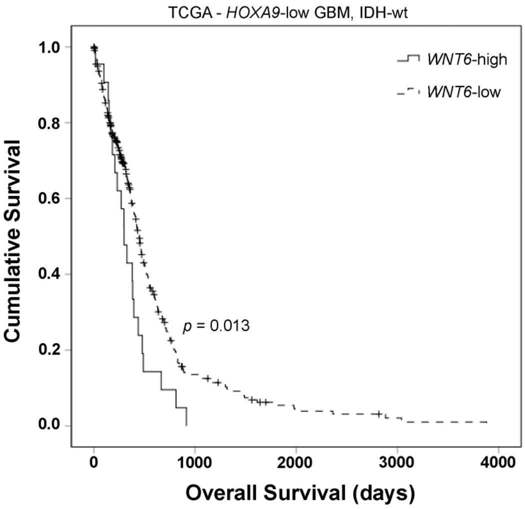
TCGA - HOXA9-low GBM, IDH-wt
WNT6-high
WNT6-low
p = 0.013
Cumulative Survival
1.0
0.8
0.6
0.4
0.2
0.0
0
1000
2000
3000
4000
Overall Survival (days)
